# The complete mitochondrial genome of the maize weevil, *Sitophilus zeamais* (Coleoptera: Curculionidae)

**DOI:** 10.1080/23802359.2020.1800426

**Published:** 2020-08-03

**Authors:** Jinhong Zhao, Wei Xu

**Affiliations:** Department of Parasitology, Wannan Medical College, Wuhu, China

**Keywords:** *Sitophilus zeamais*, curculionoidea, mitogenome, phylogenetic analysis

## Abstract

The complete mitochondrial genome (mitogenome) of *Sitophilus zeamais* was determined by an Illumina platform. This mitogenome was 18,531 bp in length, containing 13 protein coding genes, 2 rRNA genes, 22 tRNA genes that is typical among curculionoidea. Stop codon was incomplete for ND4 gene and ND5. The non-coding intergenic regions have increased in size to 1033 bp due to expansion of tandem repeat arrays. Phylogenetic analysis on mitogenome of *S. zeamais* would further contribute to resolve phylogenetic position and interrelationships of *Sitophilus*.

*Sitophilus zeamais* (Motschulsky, 1855) (Coleoptera: Curculionidae), which is a common pest of stored product and has a worldwide distribution, cause the greatest levels of damage to stored grain and threaten the food security (Ojo et al. 2016). There have been many reports on biological characteristics, seasonal dynamics and biological control of the weevils (Haddi et al. 2018; Prates et al. 2019). However, genetic characteristics can be applied to phylogenetic analyses to estimate the evolutionary genomics based on comparisons of the mitochondrial genomes (Zhao et al. 2018; Wang et al. 2019). In this study, the specimens of *S. zeamais* were collected from Baoding (115 46′E, 38 88′N), Hebei province, China in 2019, and were stored in the Department of Parasitology, Wannan Medical College, Anhui Province, China (No: WNMC-I-107). We sequenced and annotated the complete mitogenome of *S. zeamais* by an Illumina platform. The mitogenome data of *S. zeamais* was determined and deposited to the GenBank DNA databases with accession number MT294139.

The mitogenome of *S. zeamais* was 18,531 bp in size and consists of 13 protein-coding, 22 tRNA genes and 2 rRNA genes. All genes were encoded on the the heavy strand (H) except for four protein-coding gene (ND5, ND4, ND4L and ND1), eight tRNAs (tRNA-Gln, tRNA-Cys, tRNA-Tyr, tRNA-Phe, tRNA-His, tRNA-Pro, tRNA-Leu2 and tRNA-Val) and two ribosomal RNAs (rrnL and rrnS). The overall base composition of *S. zeamais* show a strong biased toward A and T nucleotides, and the AT contents of control regions were the highest (87.0%), as generally seen in other coleopteran mitogenomes (Korkmaz et al. 2016). All thirteen PCGs encoded by the *S. zeamai* mitogenomes are initiated with ATN (six ATT, five ATG and one ATA) start codon with the exception of ND1 with a TTG codon, and the complete stop codon (four TAG and seven TAA) with exception for ND5 and ND4 which use incomplete stop codon T. There have a non-coding sequence (1033 bp) between tRNA Ile and tRNA Gln, with three tandem repeats interspersed with period sizes of 105 bp. Moreover, the longest spacer region corresponds to the AT-rich control region (2832 bp) were located between rrnS and tRNA Ile.

To further understand the phylogenetic position of *S. zeamai*, the concatenated nucleotide sequences of 13 PCGs from 9 Curculionidae species and outgroup species from the family Mordellidae (*Mordella atrata*) were used for the phylogenetic analysis by the Neighbor-Joining (NJ) method (Figure 1). The NJ phylogenetic tree showed that *S. zeamai* was more closely related to *S. oryzae* than to other species, and then cluster with the *Sphenophorus* sp. These data will be useful for molecular identification and phylogenetic studies of *S. zeamai*.

**Figure 1.**
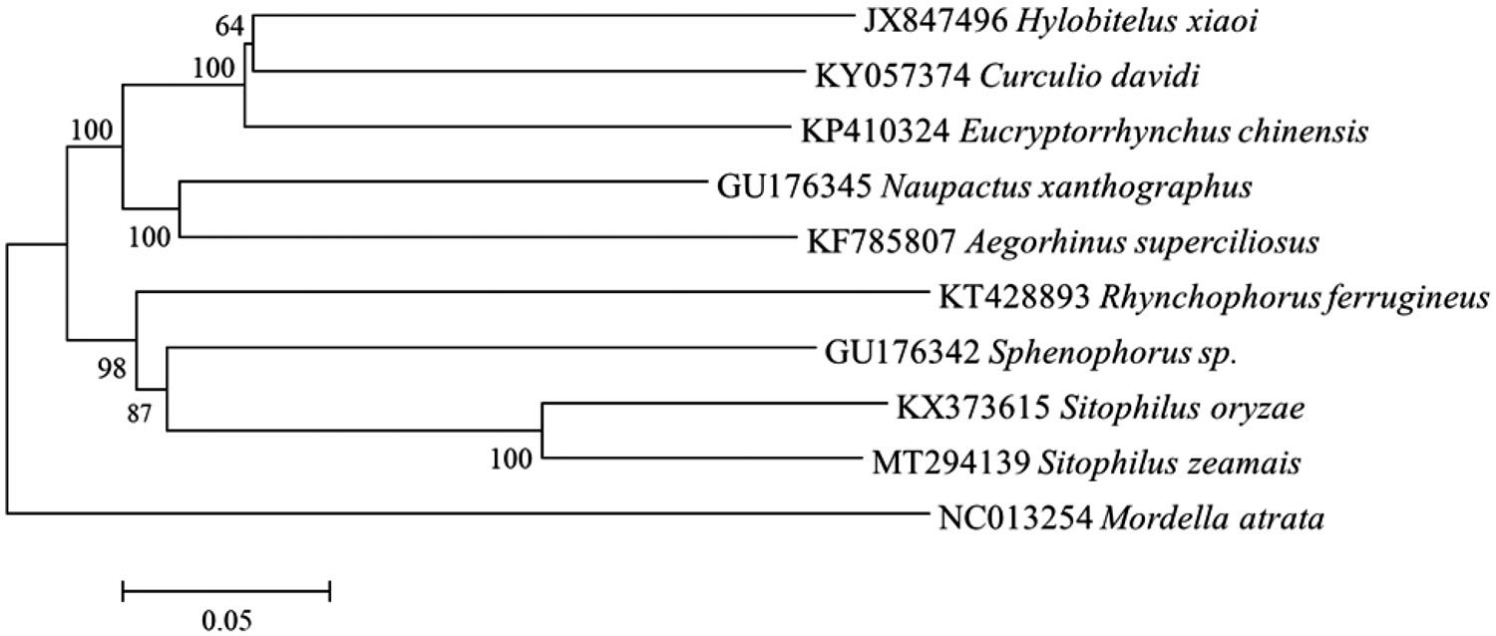
The NJ phylogenetic tree of *S. zeamai* based on the concatenated nucleotide sequences of 13 PCGs.

## Data Availability

https://www.ncbi.nlm.nih.gov/nuccore/MT294139
